# Effect of educational intervention on improving nurse’s general health in military hospitals of Iran: a quasi-experimental study

**DOI:** 10.1186/s12912-022-01032-8

**Published:** 2022-09-13

**Authors:** Vahid Ranaei, Fatemeh Kalroozi, Mojtaba Sadeghi, Soudabeh Yarmohammadi, Kristin Haglund, Nahid Mehrabi

**Affiliations:** 1 Researcher, Department of Health Information Technology, Faculty of Paramedicine, AJA University of Medical Sciences, Tehran, Iran; 2 Assistant Professor in Pediatric Nursing Department, Faculty of Nursing, AJA University of Medical Sciences, Shariati St, Kaj St, Tehran, Iran; 3 Faculty Member of Department of Health Information Technology, AJA University of Medical Science, Tehran, Iran; 4 School of Public Health and Safety, Shahid Beheshti University of Medical Sciences, Tehran, Iran; 5 College of Nursing, Marquette University, Milwaukee, WI USA; 6 Assistant Professor in Health Information Management, Paramedical School AJA University of Medical Sciences, Fathemi St, Etemad zadhe St, Tehran, Iran

**Keywords:** Military hospitals, Psychiatry, Nurse, Health, Iran

## Abstract

**Background:**

Nursing in military hospitals affects their general health. Educational interventions can help to maintain the general health of nurses. This study aimed to evaluate the effect of an educational intervention to improve the general health of nurses in military hospitals.

**Methods:**

In this quasi-experimental study, 146 nurses working in military hospitals in Tehran, Iran in 2021 were included in the study. The one-month educational intervention included videos, pamphlets, and motivational messages about promoting general health. General health questionnaire with 28 items (GHQ28) was study tool. The allocation of nurses to groups was not random, it was based on personal interest. Frequency (percentage), and mean (standard deviation) were used to describe, and the chi-square test, Fisher’s exact test, independent and paired t-test were used for data analysis. All analyzes were performed in SPSS 24 software with a significance level of 5%.

**Results:**

A total of 146 nurses participated in the study, most of them were in the age group of 30 to 40 years (64 people, 43.8%), and 76.7% (112 people) of them were women. The results of the independent sample t-test showed after the intervention, general health scores in the intervention group had a significant decrease compared to the control (*p* < 0.001) (change in intervention group = -31.1, V.S change in control = 0.55). The results of paired t-test showed that only in the intervention group, the value of the general health score and its dimensions were significantly different (*p* < 0.001).

**Conclusions:**

The educational intervention performed well and decreased the score of the general health of military nurses. Given that these positive effects may be temporary, it is necessary to design and perform educational interventions over a longer period.

## Background

The World Health Organization’s latest definition of health refers not only to the absence of disease or disability but also to the state of complete physical, mental, emotional, social, and spiritual well-being [1, 2]. Health Dimensions as a full whole health picture are inextricably interwoven, this means that if one dimension changes, the others will change as well [2].

Previous studies have pointed out there was a bidirectional relationship between health and work [3–6], so having a job can lead to increased general health. On the other hand, health status has a direct impact on job performance. Health care workers due to the sensitivity of patient care, require a high level of general health [7]. One of the most stressful occupations of the hospital is nursing, in which high levels of stress and fatigue, sleep deprivation, night shifts, life problems, and workload all affect the level of their general health [8, 9]. Nurses usually have a good level of health when they are hired, but over time their general health level decreases under job stress and workload [10].

One of the most important disorders reported in the field of the general health of nurses is depression [11]. In a meta-analysis study conducted to estimate the rate of depression and anxiety in 2022, the pooled prevalence of depression and anxiety among nurses was 22% and 29%, respectively [12]. In the coronavirus pandemic, the general health of nurses was severely affected by the increase in responsibilities and mortality of patients and family members, and colleagues [13].

During the coronavirus pandemics, various studies have been performed in different parts of the world. The prevalence of nurses’ depression in different studies was investigated for instance China with 56.2% [14], the Philippines and USA with 8.7% [15], Turkey, England, Brazil, and Canada with 4.35% [16]. The results of a review study showed most Iranian studies (62%) reported the general health level of nurses as unfavorable and reported mental disorders as the most common complication [17]. In a study in Iran, social dysfunction, anxiety and physical symptoms were the most common health disorders among nurses with a prevalence of 71.4%, 62.5%, and 60.7%, respectively [18].

Nursing in military hospitals in addition to the mentioned problems has other problems such as facing patients with severe war injuries which requires patient care by maintaining the confidentiality of their information [19, 20]. On the other hand, the environments of military hospitals are different from those of other hospitals, and due to strict military rules, the environments are a bit dry and serious, and if a nurse makes a mistake, it can have organizational and political complications [21, 22]. Therefore, the combination of nursing and working in a military hospital exacerbates the job stress and workload of nurses, this issue has been confirmed in various studies [23–25]. The effectiveness of health-related educational interventions has been emphasized in many studies and they are practical ways to promote health among nurses [26–31].

In our previous observational study of nurses in military hospitals in Tehran, 61.4%, did not have the desired level of general health [32]. To the best of our knowledge, educational intervention to improve the general health of nurses working in military hospitals has not been done. Therefore, this interventional study was conducted to investigate the effects of educational intervention on the general health of nurses working in military hospitals in Tehran, Iran.

## Methods

### Study design and setting

This study was a quasi-experimental study with non-randomized control and intervention groups [33]. Iran was engaged in an imposed war with Iraq for 8 years, many military personnel were injured and disabled, and the need for long-term treatment interventions in dedicated hospitals was felt. Therefore, military hospitals have been formed to meet the health and medical needs of soldiers injured in war and also soldiers who are injured in military exercises.

#### Participants

**Fig. 1:**
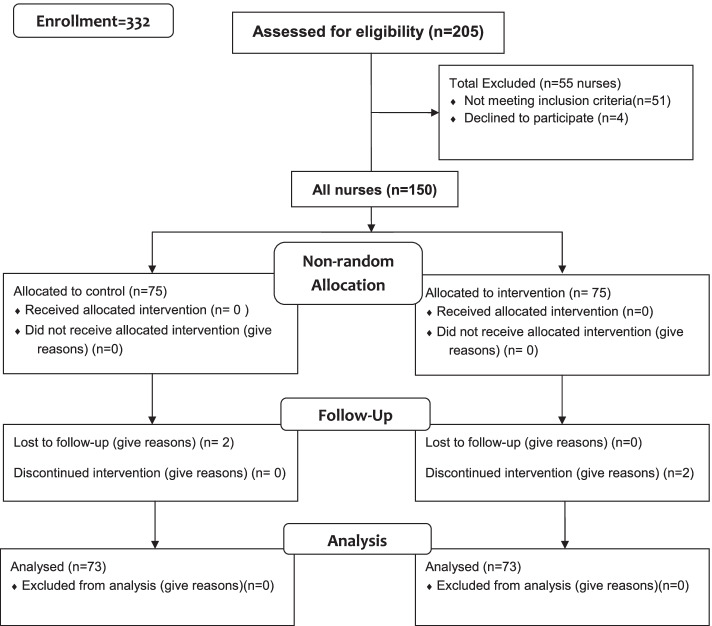
CONSORT Flow Diagram

The statistical population was all nurses working in military hospitals in Tehran. Inclusion criteria were having an insufficient level of general health based on the general health questionnaire (GHQ28 score > 24), no physical disability, and at least 5 years of work experience. Exclusion criteria were the presence of an infectious underlying disease such as coronavirus and lack of regular attendance at intervention programs. The study flow diagram is presented in Fig. 1

Random assignment of nurses to the intervention and control groups was not possible. Nurses self-selected to participate in the intervention or control group.

### Sample size and sampling procedure

The sampling method in this study was multi-staged. In the first stage, using cluster sampling, 8 military hospitals in Tehran were considered as clusters, and then 4 hospitals were randomly selected.

In the second stage using the sample size calculation formula, 1, 332 nurses through probability proportional to size cluster sampling (PPS-CS) were selected. The general health questionnaire was provided to them, then based on the cut-off point of 24 in the score of the questionnaire, nurses with disorders in their general health were identified (they were 205 people).

In the third step, according to the sample size calculated in the formula of sample size 2, by checking the inclusion criteria, 150 people through convenience sampling among the nurses with disorders (205 people) were selected. Probability proportional to size cluster sampling (PPS-CS) in the simplest definition means sampling from each cluster proportional to the population of that cluster. To calculate the required sample of each cluster, it is necessary to calculate the ratio of the population of each cluster to the total population of the selected clusters and multiply it by the total sample size [34]. Based on this, the population of nurses in Beasat, Family, 501, and Golestan military hospitals were 320, 290, 280, and 310, respectively. According to the sample size of 332, the number of nurses selected from Beasat, Family, 501, and Golestan military hospitals were 88, 80, 77, and 85.

The method of calculating the sample size in the first and second stages is provided below:

- Sample size in stage 1

Based on the general health study of Rajabzadeh et al. [35] in 2016 among nurses, the results showed 25% of them were healthy. Putting this information, in Cochran sample size formula, considering the significance level of 5% and the error rate of estimating 5 (d = 0.05), the sample size was equal to 288 people.

$${n}_{0}\ge \frac{{z}_{ 1-\frac{a}{2}}^{2}p\times \left(1-p\right)}{{d}^{2}}=\frac{{1.96}^{2}\times 0.25\times 0.75}{{\left(0.05\right)}^{2}}=288$$

With considering 13% probability of non-response rate, the final sample size of at least 332 people was obtained.

- Sample size in stage 2

To determine the sample size at this stage, the intervention study of Amini et al. [36] which examined the effect of communication skills training on the general health of nurses, was used. According to the study, the general health score in the control group had a mean (standard deviation) of 38.25 (7.95) and in the intervention group was 55.65 (9.26). Using this information and placing it in the sample size formula and considering the significance level of 5% and the test power of 80%, the sample size of 64 people in each group was estimated.

$${n}_{0}\ge \frac{\left(1+k\right)}{k}\frac{{\left({z}_{1-\frac{a}{2}}+{z}_{1-\beta }\right)}^{2}}{{d}^{2}}+\frac{{{z}_{1-\frac{a}{2}}}^{2}}{2\left(1+k\right)};k=1, a=0.05,d=1.3\Rightarrow n\ge 64$$

Then, considering the 15% non-response rate, and equal sample size in two groups (k = 1), the final sample size of 75 people in each group was estimated.

In the present study, after confirming the face and content validity of GHQ28 by experts, the internal consistency was recalculated by Cronbach’s alpha, which was equal to 0.75. Also, stability reliability was measured by measuring general health twice with a time interval of 2 weeks by calculating the intra-cluster correlation (ICC) coefficient, which was found to be 0.78.

### Educational intervention

Educational materials were provided to participants electronically through the WhatsApp and Telegram apps. The nurses used their personal phones. They were free to choose the type of application to receive educational content.. During the intervention, SMS reminders were continuously sent to them. In order to prevent the transfer of the content of the educational intervention during the intervention, all the participants of the intervention group were initially promised not to publish the educational information until the end of the study. The educational interventions during one month were as following:

1. Playing 4 educational videos of approximately 3 min in the form of a researcher’s lecture and using appropriate photos and animations containing strategies to improve the general health of nurses. Each week, one of these 4 educational videos for the target population was broadcasted on social media (WhatsApp and Telegram).
2. Sending motivational text messages to the mobile phones of the nurses in the intervention group for 30 days. In this way, a new training message was sent to the intervention group every day and this message was repeated 4 times during the day. These educational videos and motivational text messages were designed to maximize the general health in nurses.
3. Designing a 25-page educational booklet entitled “Educational booklet” and an educational pamphlet to enhance the level of nurse’s general health. The educational pamphlet was provided to the nurses in both electronic and printed form and they were requested to read the pamphlet carefully.

To ensure that participants had read the study material, participants were contacted by phone and asked if they had read the educational content.

#### Data collection

Data were collected electronically. All educational content, as well as questionnaires, were provided to the participants through social media such as WhatsApp and Telegram.

The duration of the study was six months. Pre-test questionnaires were completed by the intervention and control groups. Then the educational intervention was implemented for one month for the intervention group. One month after the educational intervention, an educational reminder session was implemented for the intervention group. Four months after the reminder session, the post-test questionnaires were completed by the participants in the intervention and control groups.

#### Data analysis

Frequency (percentage), mean and standard deviation were used to describe the variables. The normality of health scores was assessed by the Kolmogorov–Smirnov test. Chi-square test, Fisher’s exact test, and independent samples t-test were used to evaluate the homogeneity of study groups in terms of baseline variables. To compare between-group and within-group health scores before and after the intervention, an independent sample t-test and paired sample t-test were used. The Cohen-d method was used to calculate the effect size. In this method, for independent groups due to the equal size of the groups, it is enough to subtract the means and divide by the pooled standard deviation, for the dependent groups we also need to enter the correlation between two measurements. All analyzes were performed in SPSS 24 software with considering a significance level of 5%.

## Results

A total of 146 nurses participated in the study, most of them were in the age group of 30 to 40 years (64 people, 43.8%), 76.7% (112 people) were women, 78.8% (115 people) were married, and most of them had a bachelor’s degree (52.1%, 76 people). In terms of age, most of the participants in the intervention and control groups were in the age group of 30 to 40 years (46.6% vs. 41.1%). In terms of gender, women were the dominant population in both intervention and control groups (80.8% vs. 72.6%). Most of the nurses in the intervention and control groups were married (82.2% vs. 75.3%). In terms of educational level, most nurses in the intervention and control groups had a bachelor’s degree (53.4%, vs. 50.7%). Most people in the intervention group had a work experience of 10 to 15 years (34.2%) and in the control group most had 5 to 10 years of work experience (30.1%). In the control group, the nurses’ workplace ward was mostly chemotherapy (21.9%) and in the intervention group, was mostly surgery (23.3%).The results of the normality test through the Kolmogorov–Smirnov test showed that general health scores in the first and second measurements had a normal distribution (*p* > 0.05).

The results of the homogeneity evaluation of study groups in terms of baseline variables showed that the two groups were not significantly different in terms of age, gender, marital status, education, work experience, workplace hospital, and specialized work ward (*p* > 0.05) (Table 1). According to Table 1, the results of Fisher’s exact test showed that the two groups were not significantly different in terms of age, work experience, and workplace (*p* > 0.05).

**Table 1:** Results of evaluation of homogeneity of study groups in terms of Baseline variables

Variable	Levels	Control		Intervention		*P*
		N	%	N	%	
**Age**	20–30	8	11	4	5.5	0.435*
	30–40	30	41.1	34	46.6	
	40–50	29	39.7	32	43.8	
	50–60	6	8.2	3	4.1	
**Gender**	Male	20	27.4	14	19.2	0.24**
	Female	53	72.6	59	80.8	
**Marital status**	Single	18	24.7	13	17.8	0.31**
	Married	55	75.3	60	82.2	
**Education level**	BSc	37	50.7	39	53.4	0.74**
	MSc	36	49.3	34	46.6	
**Work experience**	< 5	5	6.8	2	2.7	0.495*
	5–10	22	30.1	24	32.9	
	10–15	18	24.7	25	34.2	
	15–20	20	27.4	16	21.9	
	20–25	3	4.1	4	5.5	
	25–30	5	6.8	2	2.7	
**Hospital Name**	Beasat	21	28.8	19	26	0.858**
	501	19	26	18	24.7	
	Family	22	30.1	21	28.8	
	Golestan	11	15.1	15	20.5	
**Workplace ward**	Internal	7	9.6	13	17.8	0.68*
	Intensive Care	2	2.7	2	2.7	
	Obstetrics and Gynecology	6	8.2	3	4.1	
	Surgery	12	16.4	17	23.3	
	Operating room	7	9.6	6	8.2	
	Chemotherapy	16	21.9	15	20.5	
	Para clinical and diagnostic	7	9.6	4	5.5	
	Outpatient	3	4.1	1	1.4	
	Neurology	9	12.3	6	8.2	
	Neonatal and pediatric	4	5.5	6	8.2	

^*^ Fisher’s exact test

^**^ Chi square test

The results of measuring general health on baseline and after the intervention are presented in Table 2. According to Table 2, the results of the independent samples t-test showed that in baseline measurement, the mean scores of general health in the two groups were not significantly different (*p* > 0.05). After the intervention, general health scores had a significant decrease in the intervention group than control (*p* < 0.001) indicating that the intervention group had improved general health following the intervention. The results of paired t-test in the intra-group comparison also showed that only in the intervention group, changes in somatic symptoms, anxiety/insomnia, social dysfunction, severe depression, and general health were statistically significant (*p* < 0.001) (Table 2).

**Table 2:** The effect of educational intervention based on the theory of planned behavior on improving Nurse’s general health

Variable	Group	Pre-intervention		Post-intervention		P*	Effect size (correlation)
		Mean	SD	Mean	SD		
**Somatic symptoms**	Intervention	10.40	0.52	2.92	0.92	< 0.001	-11.65(0.24)
	control	10.68	1.52	10.66	1.30	0.861	-0.021(0.56)
	P**	0.128		< 0.001			
	Effect size***	-0.26		-6.78			
**Anxiety/Insomnia**	Intervention	10.89	0.97	3.22	1.12	< 0.001	-5.27(-0.1)
	control	11.00	1.08	11.01	1.50	0.933	0.08(0.46)
	P**	0.519		< 0.001			
	Effect size	- 0.10		-5.91			
**Social dysfunction**	Intervention	10.52	0.65	2.88	0.96	< 0.001	-8.58(0.06)
	control	10.55	1.08	10.29	0.87	0.058	-0.20(0.32)
	P**	0.853		< 0.001			
	Effect size	-0.02		-8.13			
**Depressive symptoms**	Intervention	10.66	0.80	2.89	1.02	< 0.001	-6.62(-0.003)
	control	10.63	0.99	10.45	1.85	0.470	-0.13(0.002)
	P**	0.855		< 0.001			
	Effect size	0.02		-4.49			
**General Health**	Intervention	42.82	3.07	11.72	2.89	< 0.001	-7.33(-0.09)
	control	43.07	2.76	42.52	3.23	0.222	-0.15(0.23)
	P**	0.611		< 0.001			
	Effect size	-0.08		-10.06			

^*^ Paired sample t-test

^**^ Independent sample t-test

^***^ Cohen’s d

**Fig. 2:**
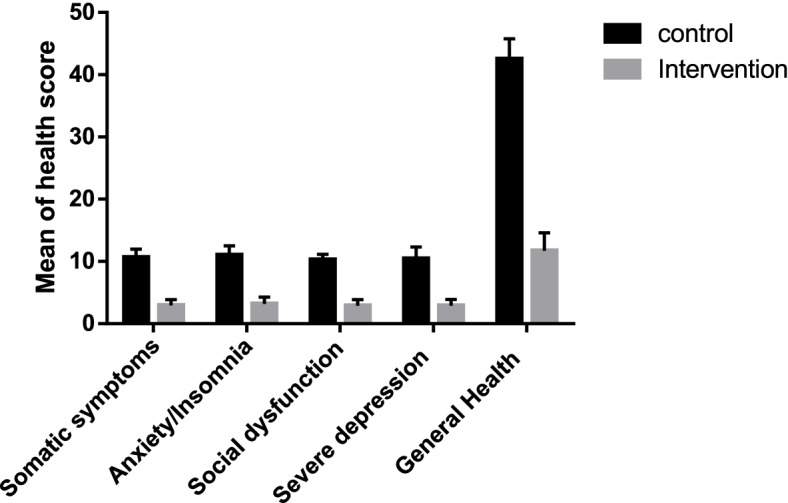
Comparison of health score in two groups

As shown in Fig. 2, the general health scores of the intervention group were significantly lower than the control group.

## Discussion

The purpose of this study was to design and test an electronic health intervention on the general health of nurses working in military hospitals in Tehran, Iran.

In accordance with this study, other studies have used interventions to raise awareness about existing solutions for health care workers that could assist with their general health care during this pandemic. For example, Rastegar et al. used social media platforms. The questionnaires were delivered to assess the initiative during the COVID-19 pandemic. The questionnaire was based on five questions on the impact of the intervention on health care workers. 71% of participants believed the platform had a significant impact on helping them adjust faster to the situation [37].

Blake et al. used an evidence-based e-learning package on the psychological well-being of health care workers. 82% of health care workers stated having used the information provided in the e-package in their work or home lives, and all of them anticipated they would use it in the future [38].

Chen et al., Rajkumar, and Huang et al. also have used online resources and hotlines for interventions. The health care workers in these studies were reluctant to participate in individual psychology interventions provided to them, even though they were showing signs of psychological issues. They had strong concerns regarding fear of affecting their families, not knowing how to deal with patients refusing to cooperate with medical and safety measures, shortage of protective equipment, lack of rest, and dealing with patients’ anxiety and panic [39–41].

Somatic symptoms are a dimension of health that include no pain, weakness, shortness of breath, or the absence of any physical symptoms that lead to functional problems [42]. In the present study, general health was assessed from the perspective of the nurse’s somatic symptoms. Our results showed that nurses’ physical symptoms in the intervention group improved significantly after the educational intervention. Therefore, in the intervention group, a significant decrease in the mean score of this dimension was obvious (see Cohen’s d in Table 2). Gandhi et al. 2014 in a study of nurses’ general health, found that the complication of physical symptoms in nurses was directly related to their perceived stress and negatively related to perceived job satisfaction [43]. In the present educational intervention, one of the goals was to present a correct picture of the disorders and to create a correct understanding of the health-threatening factors, which ultimately improved the level of physical symptoms of nurses in the intervention group.

The difficulty in falling asleep and waking up or waking up with fatigue and pain all refer to the complication of insomnia [44]. Some of the most important causes of insomnia include stress, jet lag, drug and coffee consumption, anxiety, and depression [45]. Nurses are prone to insomnia due to shift working [46]. The results of our study showed that the educational intervention was effective in reducing the insomnia rate in the intervention group compared to the control group. Studies have shown that insomnia is directly related to anxiety and stress [47, 48]. In military hospital nursing, caring for patients is stressful in terms of security and politics, because in case of human error, it may be considered as a political goal and several troubles might happen for the nurse [21]. The educational intervention of the present study also reduced anxiety among the nurses in the intervention group. Papa et al. 2020 in a meta-analysis study on nursing studies, reported that the pooled prevalence of anxiety, depression, and insomnia was 23.2%, 22.8%, and 38.9%, respectively [12]. They also noted a strong association between insomnia, anxiety, and depression. Therefore, to solve the problem of insomnia in nurses, educational interventions must also consider the two factors of anxiety and depression, which fortunately in the present study, educational intervention included recommendations to control these two factors.

Social dysfunction in the case of nurses means the inability to establish healthy social relationships and social adjustment, lack of altruism, lack of sense of social responsibility [49]. The results of our study showed that educational intervention in the intervention group had significantly improved social dysfunction (high Cohen’s d value). In a study in 2021, Wang et al. examined the factors associated with social dysfunction and identified fatigue, depression, and lack of friends as the most effective factors in social dysfunction [50]. Since fatigue and depression were reported in most studies in nurses, so in the present educational intervention, which improved the level of social inefficiency in nurses, special attention was paid to these two factors.

Depression is common among nurses and is the source of many other complications in nurses [12]. The results of the present study showed the effectiveness of educational intervention and reduction of depression score in the intervention group (high Cohen’s d value). During the preparation of the educational intervention in our study, special attention was paid to depression, so this was the success factor of our intervention. Many studies have emphasized the root cause of depression and a turning point in nurses’ health [10, 12, 17], so the emphasis of our educational intervention was on this factor. In the COVID-19 pandemic, depression increased significantly. For example, 56% of Chinese nurses had depression. Attention to depression is an important factor in general health of nurses [10]. Consistent with the present study, in 2019 a study conducted by Björkroth et al. emphasized the effectiveness of educational interventions on depression [51]. Therefore, when designing an educational intervention, it seems necessary to pay attention to depression as an important factor in the general health of nurses.

The results of our study showed that the general health score after the intervention was significantly reduced in the intervention group (high Cohen’s d value). The general health score is strongly influenced by the scores of its dimensions, so with the improvement of the situation in each dimension, the general health situation also improved. The general health of nurses is a complex issue and its temporary control is unreliable. Many studies have pointed out that the effects of the intervention are temporary and that fixed and longer interventions may be appropriate to improve the condition of nurses [26, 27, 51–53].

### Limitations and strengths

One of the limitations of this study was the difficulty of sampling and obtaining nurses information in military hospitals, due to security and political constraints in military hospitals, conducting any research requires the approval of several organizations. So it took a long time to get the approval of the relevant organizations. The impossibility of random assignment of individuals to study groups, which changed the study design to quasi-experimental.

Assessing the health status of nurses in military hospitals and conducting educational intervention was one of the strengths of this study because, to the best of our knowledge, similar study was not conducted in military hospitals and most of the studies were conducted in regular hospitals. Due to the specific and unique characteristics of military hospitals, the results of this study may not be generalizable to nurses in non-military hospitals and other healthcare settings.

## Conclusion

Nurses in military hospitals are at risk of deteriorating general health. In our study, the educational intervention performed well and was able to bring the general health of nurses to a desirable state. Although these positive effects may be temporary, it may be necessary to design and perform educational interventions with a longer duration.

## Data Availability

The data that support the findings of this study are available from corresponding author (Nahid Mehrabi) but restrictions apply to the availability of these data, which were used under license for the current study, and so are not publicly available. Data are however available from the corresponding author (Nahid Mehrabi) upon reasonable request and with permission of AJA University of Medical Sciences.

## Abbreviations

AJA: Medical school of Islamic Republic of Iran Army
GHQ-28: The General Health Questionnaire28 items
